# Genomic Surveillance Detection of SARS-CoV-1–Like Viruses in Rhinolophidae Bats, Bandarban Region, Bangladesh

**DOI:** 10.3201/eid3108.250071

**Published:** 2025-08

**Authors:** Christopher Bradburne, Ausraful Islam, Ian Bird, Elliott Abbott, Sarah Harrison, Morgan Chunn, Diana Radune, Md Rakib Hasan, Brian Janes, Sean Lovett, John Lagergren, Timothy O’Hanlon, Konad Debnath, Clifton McKee, Mohammad Enayet Hossain, Molly Gallagher, Daniel Jacobson, Mohammed Ziaur Rahman, Katie Caviness, Raina K. Plowright, Emily S. Gurley

**Affiliations:** Johns Hopkins Applied Physics Laboratory, Laurel, Maryland, USA (C. Bradburne, I. Bird, S. Harrison, M. Chunn, T. O’Hanlon, M. Gallagher); icddr,b, Dhaka, Bangladesh (A. Islam, M.R. Hasan, K. Debnath, M.E. Hossain, M.Z. Rahman); National Bioforensic Analysis Center, National Biodefense Analysis and Countermeasures Center, Fort Detrick, Maryland, USA (E. Abbott, D. Radune, B. Janes, S. Lovett, K. Caviness); Biosciences Division, Oak Ridge National Laboratory, Oak Ridge, Tennessee, USA (J. Lagergren, D. Jacobson); Johns Hopkins University Bloomberg School of Public Health, Baltimore, Maryland, USA (C. McKee, E.S. Gurley); College of Veterinary Medicine, Cornell University, Ithaca, New York, USA (R.K. Plowright)

**Keywords:** coronaviruses, viruses, vector-borne infections, SARS-CoV-1, rhinolophus, angiotensin converting enzyme 2, zoonoses, Bangladesh

## Abstract

We sequenced sarbecovirus from *Rhinolophus* spp. bats in Bandarban District, Bangladesh, in a genomic surveillance campaign during 2022–2023. Sequences shared identity with SARS-CoV-1 Tor2, which caused an outbreak of human illnesses in 2003. Describing the genetic diversity and zoonotic potential of reservoir pathogens can aid in identifying sources of future spillovers.

Zoonotic disease risk is influenced by various factors, including reservoir host density and distribution, pathogen prevalence, pathogen release, host/human proximity, and ability to infect and spread through spillover between species (*1*). Bats are well-known coronavirus reservoirs in Southeast Asia and are candidates for genomic surveillance for potential zoonotic transmission. In other ecosystems, climate and abiotic stressors can cause proximal shifts in bat roost sites, bringing bats into contact with domestic animals where virus spillover, including Hendra virus spillover, can occur (*2*,*3*; J. Lagergren et al., unpub. data, https://www.biorxiv.org/content/10.1101/2023.12.01.569640v1). Therefore, surveillance among bats colocated with bridging hosts are critical for defining spillover risk in any given region. We report preliminary results from genomic surveillance efforts focused on *Rhinolophus* spp. bat colonies at roost sites within the region of Bandarban, Bangladesh (Figure 1). We obtained study approval from the Bangladesh Forest Department and the Research Review Committee and Animal Experimentation Ethics Committee of the icddr,b (research protocol no. PR-20058, bat capture permit no. 22.01.0000.101.23.136.21.1088).

**Figure 1:**
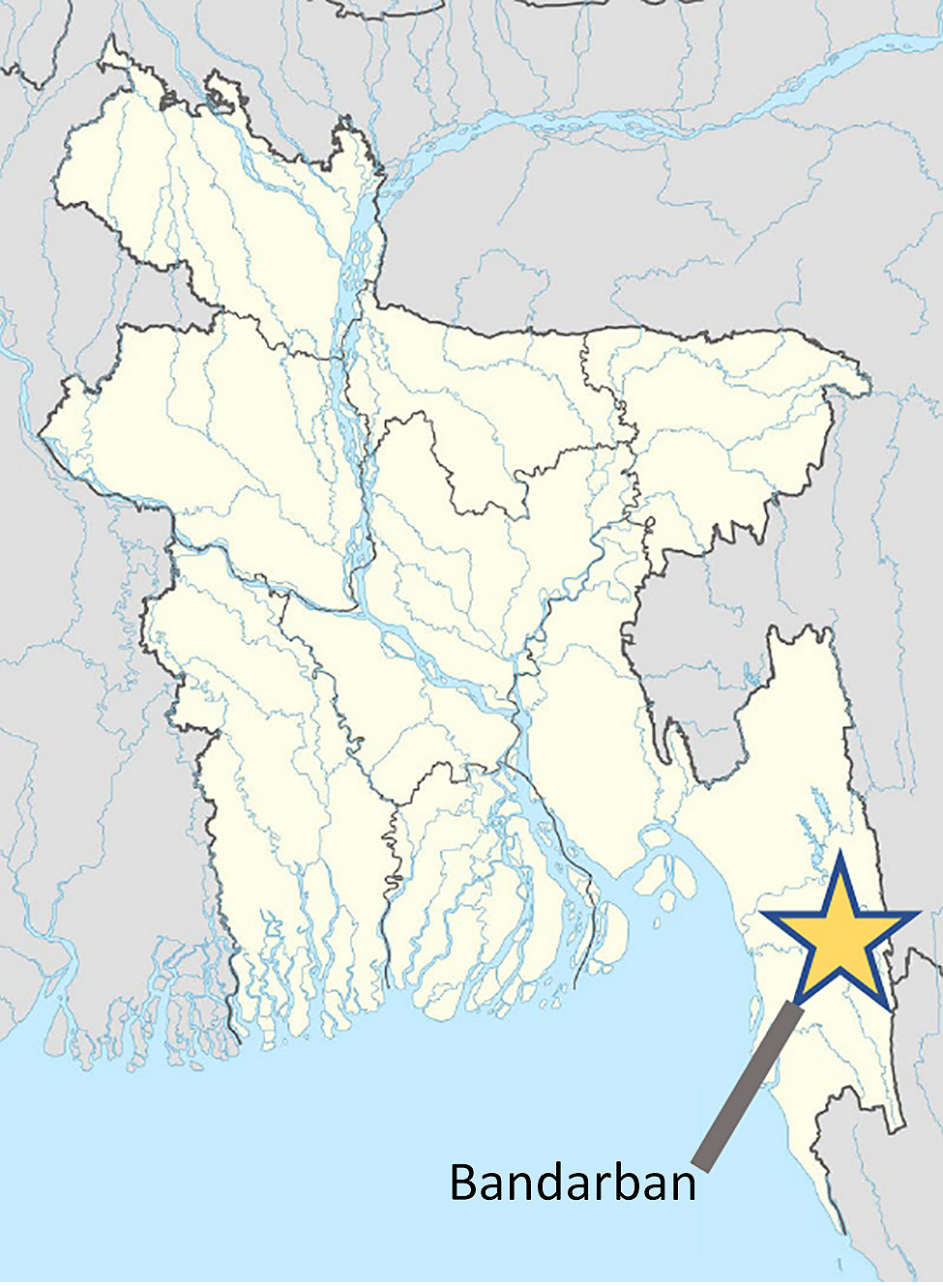
Location of Bandarban Region, Bangladesh, where genomic surveillance for detection of SARS-CoV-1–like viruses in Rhinolophidae bats was conducted during 2022–2023.

## The Study

As part of our overall field campaign, we collected fecal samples from 240 *Rhinolophus pusillus* bats, 20 each month, during May 2022–April 2023. We captured only *Rhinolophus* spp. bats and released other bat species immediately after capture. A trained veterinarian collected all the samples after anesthetizing the bats, and all the bats were released at the site of capture within 2–5 hours of capture. 

We assessed sex, weight, and health of individual bats before collecting a fecal sample and, in some cases, a blood sample from the radial vein/wing vein for immunological cell counts. For our sequencing studies, we selected fecal samples from a mix of female, male, juvenile, and adult bats with different body condition scores, choosing bats with higher leukocyte counts if those data were available. We inactivated the fecal samples by using TRIzol (Thermo Fisher Scientific, https://www.thermofisher.com) and later selected a subset of 12 fecal samples from summer 2022 (Table), representing 2 roosting locations (sites 1 and 2) in Bandarban, for initial screening on an iSeq (Illumina, https://www.illumina.com). We then performed deep sequencing on those samples by using a NextSeq 500 (Illumina). We enriched libraries by using the Comprehensive Viral Research Panel (Twist Bioscience, https://www.twistbioscience.com) supplemented with a custom-designed Chiropteran virus enrichment panel containing 134,000 probes. All sequencing was performed in a US Biosafety Level 4 facility. We deposited data into the National Center for Biotechnology Information Sequence Read Archive (submission no. SUB15226189 and BioProject no. PRJNA1249517).

**Table:** Subset of samples selected for enrichment next-generation sequencing from genomic surveillance detection of SARS-CoV-1–like viruses in Rhinolophidae bats, Bandarban Region, Bangladesh. 2022–2023*

ID no.	Sample date, 2022	Roost site	Life stage/sex	Weight, g	Leukocyte counts		Health	Sequence reads		
								Total raw, ×10^6^	Viral classified, ×10^6^	Coronavirus classified
Neutrocytes	Lymphocytes
B1	May 19	1	Adult/M	6.01	NA	NA	Fair	22.42	4.28	0
B2	May 21	1	Adult/F	5.9	NA	NA	Fair	29.2	6.69	0
B3	Jul 20	2	Adult/M	13.82	NA	NA	Good	30.16	1.27	1,420
B4	Jul 21	2	Adult/F	11.95	7	4	Good	17.97	4.01	220,000
B5	Jul 23	2	Adult/M	11.58	3	1	Good	25.9	5.54	0
B6	Jul 23	2	Juvenile/F	5.3	15	6	Fair	26.56	5.91	1,400
B7	Aug 29	2	Juvenile/M	4.69	50	3	Fair	23.76	4.99	0
B8	Aug 29	2	Juvenile/M	5.66	6	7	Fair	24.41	3.86	0
B9	Aug 29	2	Adult/F	10.86	10	12	Good	25.2	2.07	0
B10	Aug 30	2	Adult/F	11.92	25	20	Good	25.04	5.71	0
B11	Aug 30	2	Juvenile/F	5.17	10	7	Fair	28.2	7.11	0
B12	Sep 19	2	Juvenile/F	5.06	9	5	Fair	23.86	5.2	0

*In addition to neutrophils and lymphocytes shown in the table, leukocyte counts included eosinophils, basophils, and monocytes; however, none had >2 counts for any individual bat. Health was assessed by palpation of pectoral muscle mass. Viral-classified reads were compared with a viral sequencing database. ID, identification; NA, not applicable.

We identified coronavirus sequence reads in 3 samples: B3, B4, and B6. The strongest signal was in B4, which comprised 1.2% of total reads and 5.5% of classified reads in the entire B4 sample (Table; Appendix Figures 1–3). We characterized virome components by using KRAKEN2 (*4*) and RefSeq viral database version April 2023 (Illumina). The B4-derived coronavirus sequences initially had 76.7% BLASTn (https://blast.ncbi.nlm.nih.gov/Blast.cgi?PROGRAM=blastn&PAGE_TYPE=BlastSearch&LINK_LOC=blasthome) identity to SARS-CoV-1 Tor2 (Appendix Figure 4), which was isolated in 2003 from a patient who traveled from Hong Kong, China, to Toronto, Ontario, Canada, and who was hospitalized with febrile respiratory illness (*5*). 

We further compared B4 to Tor2 and relatives by creating similarity plots in SimPlot++ version 1.3 (https://github.com/Stephane-S/Simplot_PlusPlus), and creating maximum-likelihood phylogenetic trees aligning the whole genome, RNA-dependent RNA polymerase, and spike sequences by using MAFFT version 7.508 (https://mafft.cbrc.jp/alignment/software) and IQ-TREE version 2.3.6 (http://iqtree.cibiv.univie.ac.at) (Appendix Figures 4, 5) (*6*–*11*). We found 2 notable dropouts in the alignment to SARS-CoV-1 Tor2: a 1-kb gap at nonstructural protein (NSP) 2 and a 2.1-kb gap over most of the spike receptor-binding domain (RBD) (Appendix Figure 4). To obtain complete genome coverage, we designed 2 primer pairs for each gap and used those primers to generate and sequence amplicons (Appendix Table 1). Initial amplicon analysis using BLASTn provided GenBank accession no. KY417143.1, bat SARS-like coronavirus isolate RS4081, which shared 85%–94% identity over a 99% query length. The main difference was the spike region (85% identity), which had no hits for 214 nt. A BLASTx (https://blast.ncbi.nlm.nih.gov/Blast.cgi?PROGRAM=blastx&PAGE_TYPE=BlastSearch&LINK_LOC=blasthome) query of the spike-specific amplicon indicated 76.2% identity (182 mismatches and 8 gaps) over a 97% query length to GenBank accession no. QVN46559.1, a spike glycoprotein from bat SARS-like coronavirus Khosta-1. A top BLASTx hit for the NSP2-specific amplicon was protein sequence NP_828861.2, an NSP2 of SARS-CoV-1 Tor2, which had 72.5% identity over a 99% query length. Further phylogenetic analysis of whole genomes, spike, and RNA-dependent RNA polymerase supported a novel virus with close spike homology to *Sarbecovirus* spp. (Appendix Figure 5).

We used AlphaFold modeling (European Molecular Biology Laboratory, European Bioinformatics Institute, https://alphafold.ebi.ac.uk) to compare the RBD of B4 with SARS-CoV-1 Tor2 RBD (Appendix Figure 6). Folding indicated similar shape and functionality and exhibited nonsynonymous substitutions and insertions. Two insertions were asparagine dimers, located on an edge likely to interact with mammalian angiotensin converting enzyme 2 (ACE2), and 1 insertion was a threonine located on another edge, making the B4 RBD sample structurally close to a sample from a known zoonotic human outbreak.

**Figure 2:**
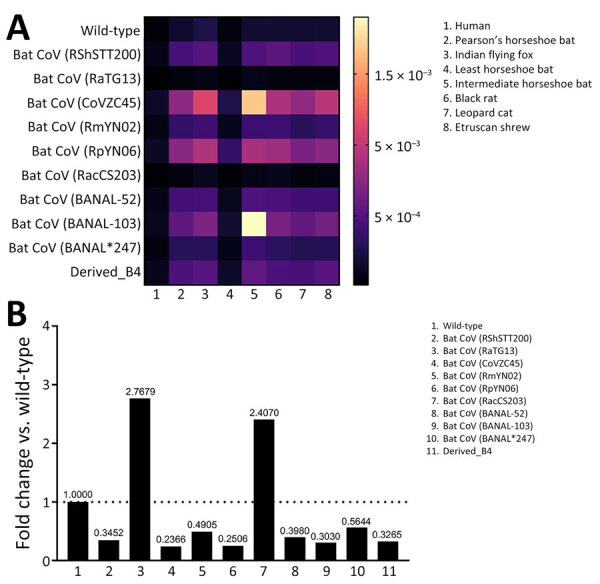
Binding efficiency and fold change of SARS-CoV-1–like virus viruses in Rhinolophidae bats, Bandarban Region, Bangladesh. A) Heatmap depicting binding efficiency of receptor binding domain (RBD) sequences from B4 and regional bridging hosts. The map shows that derived B4 RBD has low-to-moderate binding efficiency to angiotensin converting enzyme 2 (ACE2) sequences from regional bridging hosts. B) Fold change of coronavirus RBDs including derived B4 to human ACE2, relative to wild-type virus (horizontal dotted line). B4, samples from bat 4; CoV, coronavirus.

The binding of virus RBD to the primary receptor ACE2 is necessary for spillover infection to occur. We used a synthetic Förster resonance energy transfer–based assay (*12*) to test the binding affinity of known RBDs and the B4-derived RBD from our genomic surveillance data (Appendix Figure 7). We used ACE2 receptors from a variety of sympatric mammals (Appendix Table 2), including species that might reside near our bat sampling sites, such as *Rattus* spp. rats, Leopard cats (*Prionailurus bengalensis*), and humans (*13*). We chose ACE2 of the Etruscan shrew (*Suncus etruscus*) , which had sequence available for protein derivation, as a representative *Suncus* species for testing. That species has not specifically been observed yet in Bandarban, but its close relative, the *S. murinus* shrew, is widespread there and throughout Bangladesh. Dissociation constants for the novel B4 and 10 other bat coronavirus RBDs showed moderate binding of B4 to several native sympatric animals (Figure 2, panel A). 

We were also interested in the potential of the Tor2 homologous B4-derived virus to infect humans. Therefore, we evaluated binding affinity relative to the 2019 wild-type SARS-CoV-2 RBD (Figure 2, panel B). The B4-derived RBD demonstrated approximately one third the binding efficiency of the wild-type strain, which was similar to results for other tested bat coronaviruses not yet detected in humans.

In 2019, a Tor2 analog was described in a bat reservoir in Korea (*14*), indicating the viral homologue may be regionally widespread from Bangladesh to southern China and the Korean Peninsula. Abiotic stress including human land use is known to stress bat health and drive them closer to potential transitional hosts, a process implicated in spillover of other viruses (*2*,*3*).

## Conclusions

We report a coronavirus in bats in Bangladesh that has high similarity to SARS-CoV-1 Tor2, isolated in 2003 from a febrile patient who had secondary exposure to a person who contracted coronavirus from an environmental source in southern China (*5*). The virus detected in Bandarban, Bangladesh, and sequenced and analyzed in this study shares identity with Tor2, except in the NSP2 and RBD genomic regions. The synthetically expressed RBD shows moderate binding affinity to ACE2 receptors of nearby species, suggesting potential for infection of co-occurring taxa within the host range. Additional study is needed to elucidate what drives host viral shedding and if spillovers are occurring that pose a public health risk. Describing the genetic diversity and transmission potential of this and other potentially zoonotic pathogens can aid in identifying sources and risk of future emerging spillovers.

Appendix Additional information for genomic surveillance detection of SARS-CoV-1–like viruses in Rhinolophidae bats, Bandarban Region, Bangladesh.

## References

[1] Plowright RK, Parrish CR, McCallum H, Hudson PJ, Ko AI, Graham AL, et al. Pathways to zoonotic spillover. Nat Rev Microbiol. 2017;15:502–10. 10.1038/nrmicro.2017.45 28555073 PMC5791534

[2] Eby P, Peel AJ, Hoegh A, Madden W, Giles JR, Hudson PJ, et al. Pathogen spillover driven by rapid changes in bat ecology. Nature. 2023;613:340–4. 10.1038/s41586-022-05506-2 36384167 PMC9768785

[3] Cashman M, Vergara VGM, Lagergren JH, Lane M, Merlet J, Atkinson M, et al. Longitudinal effects on plant species involved in agriculture and pandemic emergence undergoing changes in abiotic stress. In: Proceedings of the Platform for Advanced Scientific Computing conference; 2023 Jun 26–28; Davos, Switzerland. New York: ACM; 2023.

[4] Wood DE, Lu J, Langmead B. Improved metagenomic analysis with Kraken 2. Genome Biol. 2019;20:257. 10.1186/s13059-019-1891-0 31779668 PMC6883579

[5] Marra MA, Jones SJM, Astell CR, Holt RA, Brooks-Wilson A, Butterfield YS, et al. The Genome sequence of the SARS-associated coronavirus. Science. 2003;300:1399–404. 10.1126/science.1085953 12730501

[6] Katoh K, Misawa K, Kuma K, Miyata T. MAFFT: a novel method for rapid multiple sequence alignment based on fast Fourier transform. Nucleic Acids Res. 2002;30:3059–66. 10.1093/nar/gkf436 12136088 PMC135756

[7] Minh BQ, Schmidt HA, Chernomor O, Schrempf D, Woodhams MD, von Haeseler A, et al. IQ-TREE 2: new models and efficient methods for phylogenetic inference in the genomic era. Mol Biol Evol. 2020;37:1530–4. 10.1093/molbev/msaa015 32011700 PMC7182206

[8] Kalyaanamoorthy S, Minh BQ, Wong TKF, von Haeseler A, Jermiin LS. ModelFinder: fast model selection for accurate phylogenetic estimates. Nat Methods. 2017;14:587–9. 10.1038/nmeth.4285 28481363 PMC5453245

[9] Hoang DT, Chernomor O, von Haeseler A, Minh BQ, Vinh LS. UFBoot2: improving the ultrafast bootstrap approximation. Mol Biol Evol. 2018;35:518–22. 10.1093/molbev/msx281 29077904 PMC5850222

[10] Samson S, Lord É, Makarenkov V. SimPlot++: a Python application for representing sequence similarity and detecting recombination. Bioinformatics. 2022;38:3118–20. 10.1093/bioinformatics/btac287 35451456

[11] Ruan YJ, Wei CL, Ee AL, Vega VB, Thoreau H, Su ST, et al. Comparative full-length genome sequence analysis of 14 SARS coronavirus isolates and common mutations associated with putative origins of infection. Lancet. 2003;361:1779–85. 10.1016/S0140-6736(03)13414-9 12781537 PMC7140172

[12] Song Y, Rodgers VGJ, Schultz JS, Liao J. Protein interaction affinity determination by quantitative FRET technology. Biotechnol Bioeng. 2012;109:2875–83. 10.1002/bit.24564 22711490

[13] Akhter T, Khan MMH, Nath S, Hasan S, Ahmed T. Photographic evidences of Pygmy white-toothed shrew *Suncus etruscus* in Bangladesh. Bangladesh J Zool. 52:337–40. 10.3329/bjz.v52i3.80794

[14] Kim Y, Son K, Kim Y-S, Lee S-Y, Jheong W, Oem J-K. Complete genome analysis of a SARS-like bat coronavirus identified in the Republic of Korea. Virus Genes. 2019;55:545–9. 10.1007/s11262-019-01668-w 31076983 PMC7089380

