# Supplementary material for: Genomic Surveillance Detection of SARS-CoV-1–Like Viruses in Rhinolophidae Bats, Bandarban Region, Bangladesh
Source: Emerg Infect Dis. 2025 Aug;31(8):1652–6. doi: 10.3201/eid3108.250071 (PMC12309761; doi:10.3201/eid3108.250071)
Supplement: Appendix — Additional information for genomic surveillance detection of SARS-CoV-1–like viruses in Rhinolophidae bats, Bandarban Region, Bangladesh. [file 25-0071-Techapp-s1.pdf]

Article DOI: https://doi.org/10.3201/eid3108.250071

EID cannot ensure accessibility for supplementary materials supplied by authors. Readers who have difficulty accessing supplementary content should contact the authors for assistance.

Genomic Surveillance Detection of SARS-CoV-1–Like Viruses in Rhinolophidae Bats, Bandarban Region, Bangladesh

Appendix

Supplementary Data

Sample Processing, Sequencing, and Analysis

For sample processing, 250 µL of each TRIzol sample was manually extracted using the Zymo Direct-zol MagBead RNA kit and eluted in 50 µL nuclease-free water. RNA was quantified with the Qubit RNA Broad Range Assay kit on Qubit Flex. Most samples were not concentrated enough to proceed with library preparation without carrier; therefore, for 10 of the samples HeLa RNA1 (10 ng, 2 µL) was added to 6.5 µL of sample and used for library input. For samples B3 and B9, 8.5 µL of sample was used for library input. RNA enrichment libraries were generated with the Illumina RNA Prep with Enrichment kit. IDT for Illumina DNA/RNA UD Indexes were used for multiplexing. For the hybridization enrichment, 200 ng of each pre-enrichment library was used, with the exception of B3 and B9 (109.43 ng and 61.125 ng, respectively). Libraries were hybridized overnight with the Twist Comprehensive Viral Panel (CVRP) spiked with a custom bat-specific probe panel designed to expand the targets of the CVRP for known bat viruses. The assay contained 134,000 probes, with both the CVRP and custom Chiropteran supplement being mixed together for one single enrichment (for more information, please contact the corresponding authors). Enriched libraries were quantified with the Agilent High Sensitivity DNA kit on the Agilent Bioanalyzer 2100. Enriched Libraries were pooled and initially sequenced on an iSeq, then deep sequenced on a NextSeq500. Sequence data were read-mapped using KRAKEN2, using two separate databases: A viral database curated and obtained from RefSeq, and a standard database containing all known microbial, viral, and eukaryotic sequences.

Hybridization Enrichment Panel

The Twist Bioscience CVRP is A1 against more than 3,000 viruses. Additionally, a custom-designed Chiropteran Supplement panel was spiked into the hybridization reaction (i.e., there was one single enrichment performed using both probe panels at the same time). The custom Chiropteran Supplement panel was designed to expand the targets of the CVRP, specifically for known bat viruses and contained 134,000 probes. For more specific information, please reach out to the corresponding authors.

Primer Walking Experiment and Sequencing

Amplicons were assembled individually and produced contigs of expected size (2417bp, 1035bp). The amplicon reads were combined with the initial iSeq and NextSeq data, and then assembled with spades generating 1 contig of 29,691 nt. These amplicons were then added to a KRAKEN2 analysis of the original B4 sample reads to generate a complete viral genome.

Appendix Table 1. Primers for CoV amplicon generation

Primer name	Genome target	Sequence (5′-3′)
1BF1	Spike	CTT TTA TTA CTT TGA TGA TGT GTT CCG CTC AGA CAC
1BF2		CTT TAT AGC CTC AGT TGC AGG TCA GCA G
3ER1		ACA TTT GCT TGA CTT GAG CAA AGA CTT CAC
3ER2		GAT CAG GCA GTA TTT GTG AAA AGT TGA AAC CAC
80EF1	nsp2	CCT TTA AAG CCA TAG TTG AGT CCT GTG GTA AC
80EF2		CAG CTT CCA CTA GTG CAT TTG TGG AAA C
2BR1		CAG GTT TTC CTT CAA CAG CAC CAG AG
2BR2		CAA GAA AGT TAG GTG ATA GAG CGC AAT ATT GC

FRET Binding Assay

To establish a method of estimating risk for a Coronavirus spillover event, a synthetic assay was employed for the evaluation of the binding affinity of SARS-CoV-2 spike Receptor Binding Domain (RBD) proteins to mammalian Angiotensin-Converting Enzyme 2 (ACEII) receptors. The idea was to be able to take any new RBDs discovered in the samples, and measure the likelihood of binding strongly to cells from potential zoonotic spillover targets. Binding was measured by affinity (the dissociation constant = Kd), with lower values (i.e., less dissociation) indicating tighter binding, and therefore a higher likelihood of establishing an infection in the mammal.

The assay, developed at JHU/APL involved synthetic synthesis of both RBDs and mammalian ACEIIs via protein expression in HEK293f cells. Expressed cells were expressed with fluorophores that, when in molecular proximity, engaged in Förster Resonance Energy Transfer (FRET), such that measurement of fluorescence intensity provided a measure of the Kd.

The calculation of fluorescence due to binding activity was calculated as described in Song et al. (*1*). The assay was carried out in 96- and 384-well formats.

Appendix Table 2. Viral receptor binding domains and host ACE2 receptors expressed and tested for binding in this study

Gene	Virus	Host species	Common name	Accession no.
		List of RBDs expressed and tested*		
S-RBD	SARS-CoV-2 (Wuhan-hu-1)	*Homo sapiens*	Human	NC_045512.2
S-RBD	SARS-CoV-2 (B.1.1.7)	*Homo sapiens*	Human	LR991698.2
S-RBD	SARS-CoV-2 (B.1.351)	*Homo sapiens*	Human	MW981442.1
S-RBD	SARS-CoV-2 (P.1)	*Homo sapiens*	Human	MZ169910.1
S-RBD	SARS-CoV-2 (B.1.427)	*Homo sapiens*	Human	MW523795.1
S-RBD	SARS-CoV-2 (B.1.617)	*Homo sapiens*	Human	MZ558086.1
S-RBD	SARS-CoV-2 (D.2)	*Homo sapiens*	Human	MZ410617.1
S-RBD	SARS-CoV-2 (B.1.525)	*Homo sapiens*	Human	OL368848.1
S-RBD	SARS-CoV-2 (AV.1)	*Homo sapiens*	Human	OU417612.1
S-RBD	SARS-CoV-2 (A.23.1)	*Homo sapiens*	Human	OL369020.1
S-RBD	SARS-CoV-2 (B.1.2)	*Homo sapiens*	Human	OL467669.1
S-RBD	Bat coronavirus (BANAL-52)	*Rhinolophus malayanus*	Malayan horseshoe bat	MZ937000.1
S-RBD	Bat coronavirus (BANAL-247)	*Rhinolophus malayanus*	Malayan horseshoe bat	MZ937004.1
S-RBD	SARS-CoV-2 (B.1.1.298)	*Neovision vison*	American mink	MT919525.1
S-RBD	Bat coronavirus (RacCS203)	*Rhinolophus acuminatus*	Acuminate horseshoe bat	MW251308.1
S-RBD	Bat coronavirus (RShSTT200)	*Rhinolophus shameli*	Shamel's horseshoe bat	GISAID: EPI_ISL_852605
S-RBD	Bat coronavirus (RaTG13)	*Rhinolophus affinis*	Intermediate horseshoe bat	GISAID: EPI_ISL_402131
S-RBD	RpYN06	*Rhinolophus pusillus*	Least horseshoe bat	MZ081381.1
S-RBD	RmYN02	*Rhinolophus malayanus*	Malayan horseshoe bat	GISAID: EPI_ISL_412977
S-RBD	PrC31	*Rhinolophus sp.*	Horseshoe bat	GISAID: EPI_ISL_1098866
S-RBD	CoVZC45	*Rhinolophus pusillus*	Least horseshoe bat	MG772933.1
S-RBD	CoVZXC21	*Rhinolophus pusillus*	Least horseshoe bat	MG772934.1
S-RBD	BANAL-103	*Rhinolophus pusillus*	Least horseshoe bat	MZ937001.1
S-RBD	SARS-CoV-2 (BA.2)	*Homo sapiens*	Human	ON330467.1
S-RBD	SARS-CoV-2 (BA.4)	*Homo sapiens*	Human	ON414623.1
S-RBD	SARS-CoV-2 (BA.2.12.1)	*Homo sapiens*	Human	ON429328.1
		List of ACE2s expressed and tested*		
ACE2		*Homo sapiens*	Human	NP_068576.1
ACE2		*Rhinolophus pearsonii*	Pearson's horseshoe bat	MT515622
ACE2		*Pteropus medius*	Indian Flying Fox	(*2*)
ACE2		*Sus scrofa*	Pig	NP_001116542.1
ACE2		*Capra hircus*	Goat	NP_001277036.1
ACE2		*Felis catus*	Domestic cat	NP_001034545.1
ACE2		*Canis lupis familiaris*	Domestic dog	NP_001158732.1
ACE2		*Ovis aries*	Sheep	XP_011961657.1
ACE2		*Bos tarus*	Cattle	NP_001019673.2
ACE2		*Bubalus bubalis*	Water buffalo	XP_006041602.1
ACE2		*Mustela lutreola biedermanni*	European mink	QNC68911.1
ACE2		*Mustela putorius furo*	Domestic ferret	NP_001297119.1
ACE2		*Paguma larvata*	Palm civets	Genbank: AY881174.1
ACE2		*Manis javanica*	Malayan pangolin	XP_017505752.1
ACE2		*Mus musculus*	House mouse	NP_081562.2
ACE2		*Nyctereutes procyonoides*	Raccoon dogs	Genbank: EU024940
ACE2		*Macaca mulatta*	Rhesus macaques	XP_014982444.2
ACE2		*Chlorocebus sabaeus*	African green monkey	XP_007989304.2
ACE2		Odocoileus virginianus	White-tailed deer	Collaborator (aligned to ELK ACE2)
ACE2		*Rhinolophus pusillus*	Least horseshoe bat	GQ999938.1
ACE2		*Rhinolophus shameli*	Shamel's horseshoe bat	MZ851782.1
ACE2		*Rhinolophus affinis*	Intermediate horseshoe bat	MT394203.1
ACE2		*Vulpes vulpes*	Red fox	XP_025842512.1
ACE2		*Suncus etruscus*	Etruscan shrew	XP_049622744.1
ACE2		*Arctonyx collaris*	Greater hog badger	MT663962.1

Gene	Virus	Host species	Common name	Accession no.
ACE2		*Neovison (Neogale) vison*	American mink	MW269526.1
ACE2		*Equus caballus*	Horse	XP_001490241.1
ACE2		*Prionailurus bengalensis*	Leopard cat	XM_043569674.1
ACE2		*Manis pentadactyla*	Chinese pangolinn	MT038416.1
ACE2		*Rattus rattus*	Black rat	XM_032890254.1
ACE2		*Rattus norvegicus*	Brown rat	NP_001012006.1
ACE2		*Gallus domesticus*	Chicken	XP_416822.3
ACE2		*Ictidomys tridecemlineatus*	Thirteen-lined ground squirrel	XM_005315994.4
ACE2		*Neosciurus carolinensis*	Eastern gray squirrel	XM_047535682.1
ACE2		*Corvus brachyrhynchos*	American crow	XM_017728394.1
ACE2		*Athene cunicularia*	Burrowing owl	XM_026849924.1
ACE2		*Tyto alba*	Barn owl	XM_042788706.1
ACE2		*Sturnus vulgaris*	Common starling	XM_014875884.1
ACE2		*Merops nubicus*	Northern carmine bee-eater	XM_008939271.1

*Although all RBDs and ACE2s listed were tested, only a subset relevant to the paper and the region is shown in Figure 2 in the main text.

References

1. Song Y, Rodgers VGJ, Schultz JS, Liao J. Protein interaction affinity determination by quantitative FRET technology. Biotechnol Bioeng. 2012;109:2875–83. PubMed https://doi.org/10.1002/bit.24564

2. Fouret J, Brunet FG, Binet M, Aurine N, Enchéry F, Croze S, et al. Sequencing the genome of Indian flying fox, natural reservoir of Nipah virus, using hybrid assembly and conservative secondary scaffolding. Front Microbiol. 2020;11:1807. PubMed https://doi.org/10.3389/fmicb.2020.01807

Name	Number of raw reads	Classified reads	Chordate reads	Artificial reads	Unclassified reads	Microbial reads	Bacterial reads	Viral reads	Fungal reads	Protozoan reads
B1_S1	22,418,297	99.5%	37%	0%	0.53%	62.4%	0.952%	12.5%	0%	0%
B10_S10	25,041,807	99.6%	33.3%	0%	0.431%	66.2%	1.01%	15.9%	0%	0%
B11_S11	28,197,133	99.5%	34.1%	0%	0.549%	65.3%	0.805%	19%	0%	0%
B12_S12	23,864,803	99.4%	34.6%	0%	0.59%	64.8%	1.14%	15%	0%	0%
B2_S2	29,204,716	99.6%	34.9%	0%	0.389%	64.7%	0.839%	16.5%	0%	0%
B3_S3	30,159,996	68.6%	3.41%	0%	31.4%	65.1%	31.6%	0.023%	0%	0%
B4_S4	17,973,593	99.6%	33.1%	0%	0.427%	66.4%	0.885%	15.7%	0%	0%
B5_S5	25,903,203	99.6%	41.3%	0%	0.43%	58.2%	0.755%	15.4%	0%	0%
B6_S6	26,562,145	99.5%	37.1%	0%	0.521%	62.4%	0.885%	16.6%	0%	0%
B7_S7	23,762,936	99.2%	34.4%	0%	0.818%	64.8%	1.16%	14.1%	0%	0%
B8_S8	24,414,554	99.6%	35.1%	0%	0.389%	64.4%	1.32%	8.09%	0%	0%
B9_S9	25,197,279	92.7%	10.8%	0%	7.33%	81.8%	8.33%	0.0605%	0%	0%

Appendix Figure 1. Sample processing, sequencing, and analysis. For sample processing, 250 µL of each TRIzol sample was manually extracted using the Zymo Direct-zol MagBead RNA kit and eluted in 50 µL nuclease-free water. RNA was quantified with the Qubit RNA Broad Range Assay kit on Qubit Flex. Most samples were not concentrated enough to proceed with library preparation without carrier; therefore, for 10 of the samples HeLa RNA1 (10 ng, 2 µL) was added to 6.5 µL of sample and used for library input. For samples B3 and B9, 8.5 µL of sample was used for library input. RNA enrichment libraries were generated with the Illumina RNA Prep with Enrichment kit. IDT for Illumina DNA/RNA UD Indexes were used for multiplexing. For the hybridization enrichment, 200 ng of each pre-enrichment library was used, with the exception of B3 and B9 (109.43 ng and 61.125 ng, respectively). Libraries were hybridized overnight with the Twist Comprehensive Viral Panel (CVRP) spiked with a custom bat-specific probe panel described in this supplementary file. Enriched libraries were quantified with the Agilent High Sensitivity DNA kit on the Agilent Bioanalyzer 2100. Enriched Libraries were pooled and initially sequenced on an iSeq, then deep sequenced on a NextSeq500. Sequence data were read-mapped using KRAKEN2, using two separate databases: A viral database curated and obtained from RefSeq, and a standard database containing all known microbial, viral, and eukaryotic sequences.

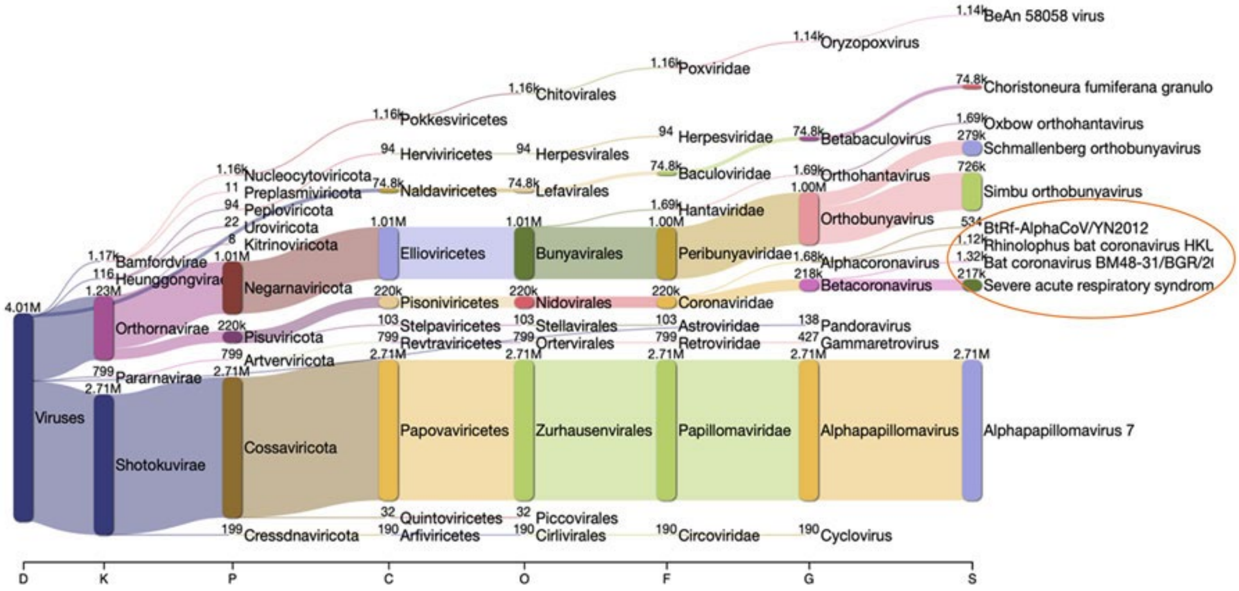

Appendix Figure 2. Virome of sample B4 and elucidation of novel bat SARS-like coronavirus genomic characteristics. Sample reads map to several closely related coronaviruses (human papillomavirus is present from the addition of 10 ng HeLa RNA added as a carrier).

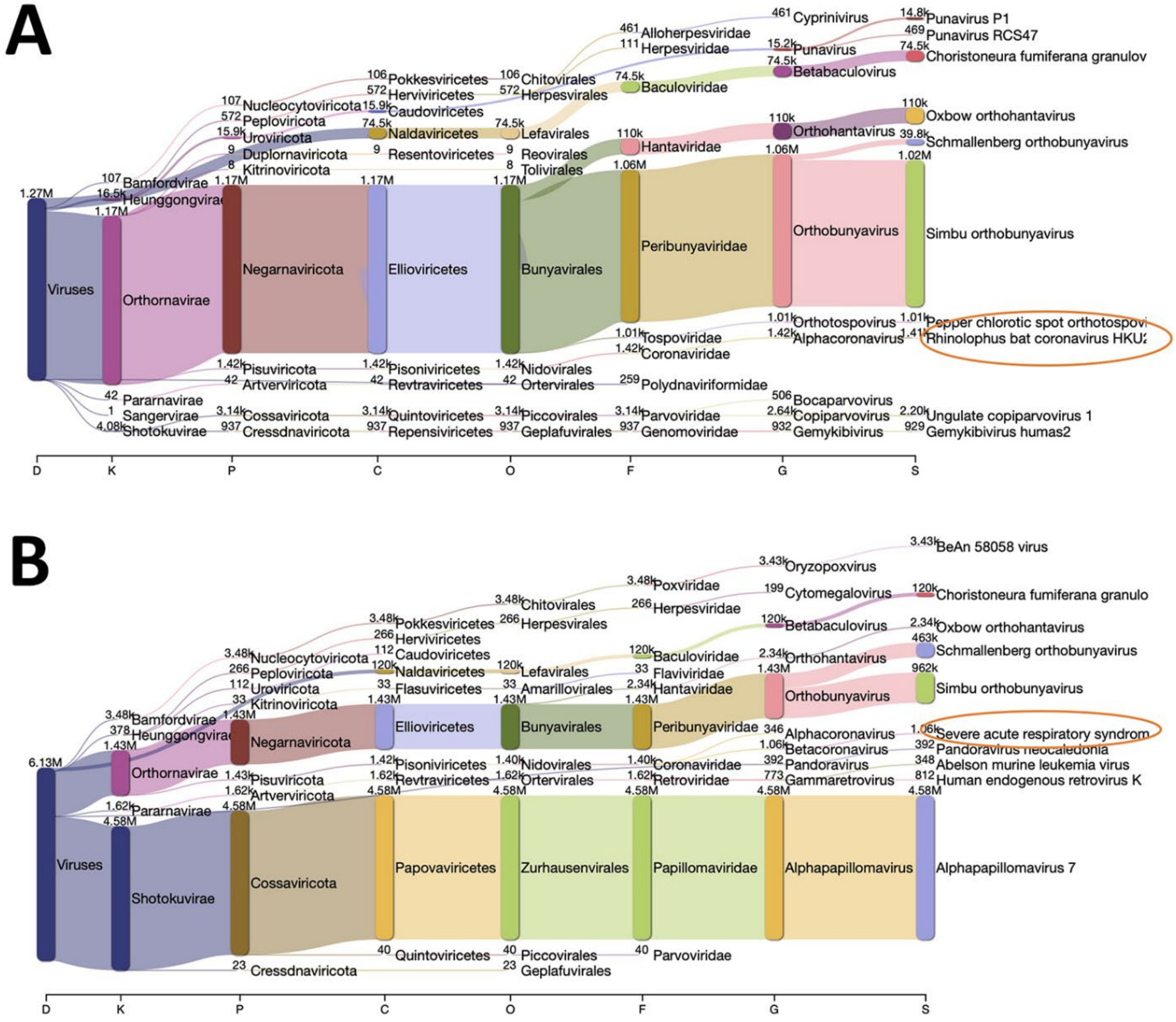

Appendix Figure 3. Viriome of samples B3 and B6 yielded sample reads mapping to coronavirus. Human papillomavirus present in sample B6 is from the addition of 10 ng HeLa RNA added as a carrier (this carrier was not added in sample B3). Read alignments for sample B3 were closest to Rhinolophus bat coronavirus HKU2, while B6 had the closest alignment to SARS Coronavirus Tor2, however both had whole genome coverage of <5% to these taxa (data not shown).

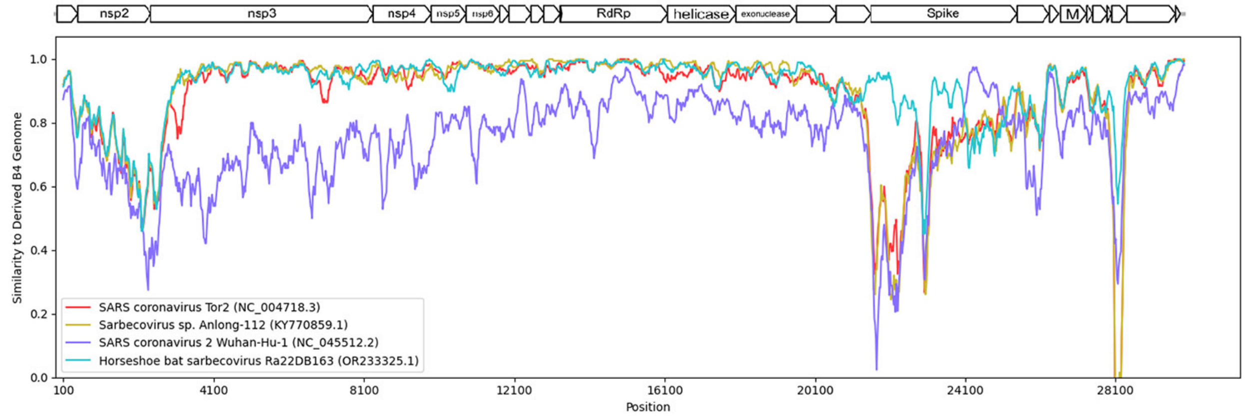

Appendix Figure 4. Sample B4 alignment of *Coronaviridae* reads is closest to 2 sarbecovirus species and SARS-CoV-1 Tor2. SARS-CoV-2 Wuhan-Hu-1 was also compared for context. Substantial dropouts (gaps) are observed at the nsp2 and viral spike protein regions. Primer walking through those dropout regions and sequencing allowed completion of the sample B4 genome. nsp, nonstructural protein; RdRp, RNA-dep endent RNA polymerase.

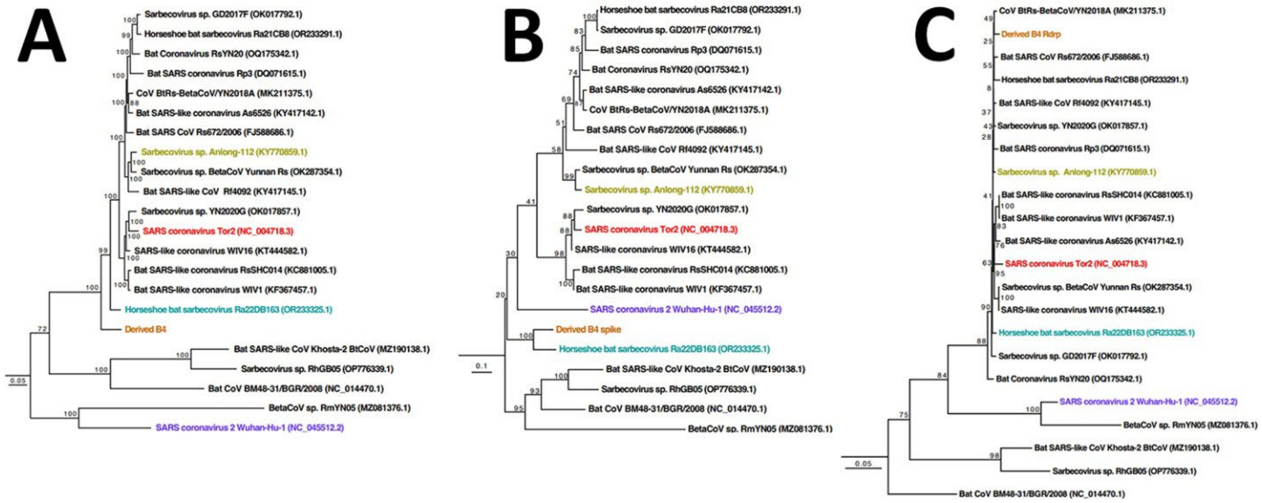

Appendix Figure 5. The completed (derived) B4 genome is closely related to Sarbecoviruses and SARS-like Coronaviruses, with a spike protein lineage that forms a unique clade with Horseshoe bat sarbecovirus Ra22DB163, but a more unique RNA-Dependent RNA Polymerase (RDRP) lineage.

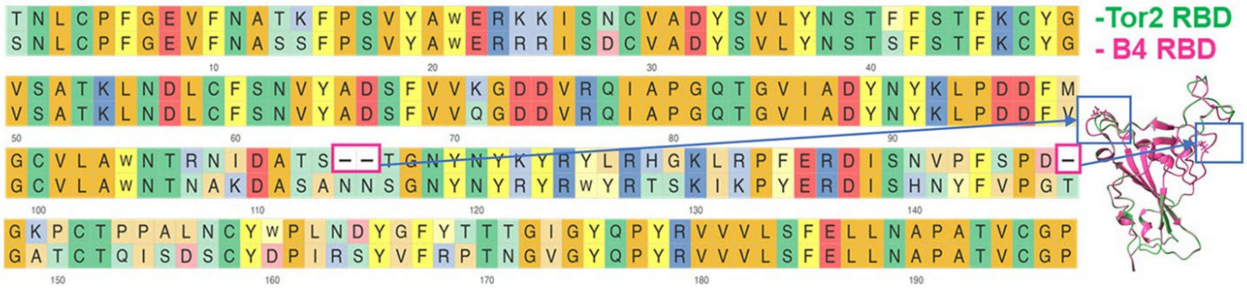

Appendix Figure 6. AlphaFold modeling of the primer-walked, sequenced spike protein region yielded similar structures to the Tor2 RBD, with single amino acid substitutions and two significant deletions.

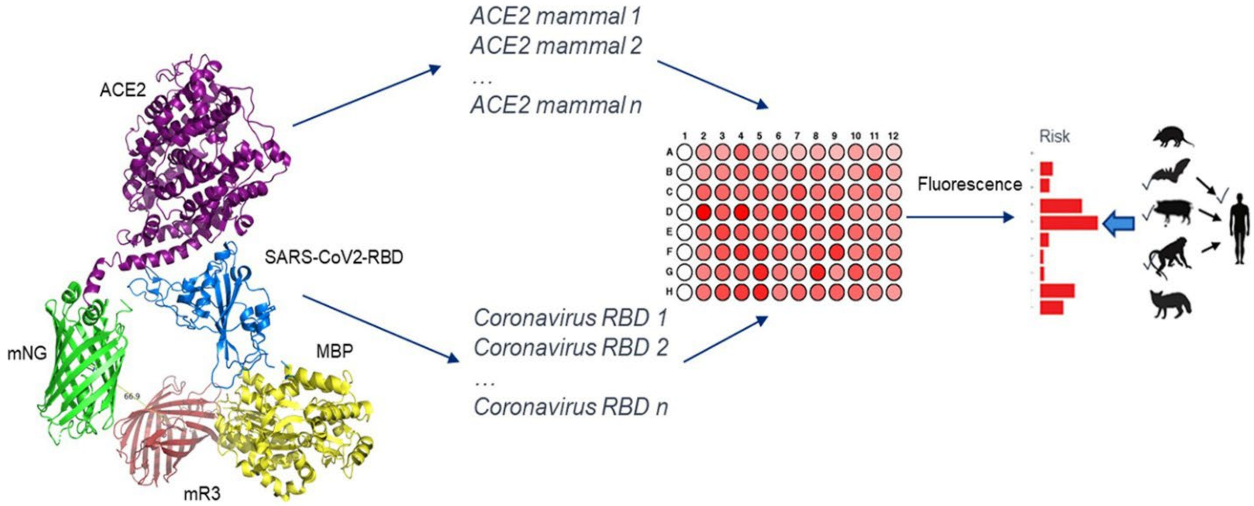

Appendix Figure 7. Binding assay employed to measure biochemical dissociation constants (Kds) of virus RBDs with mammalian ACE2 sequences to inform risk of infection. FRET interaction is measured by fluorescence, which provides a measure of virus infection risk for that particular mammal. Determination of Kd from fluorescence is given by the equation Kd = ((α)(β))/EmFRET, where EmFRET = FLDA - (α(FLDD)) – (β(FLAA)), α = unbound FLDA/unbound FLDD, and β = unbound FLDA/unbound FLAA. Excitation/Emission for each is FLDA: 485/595, FLDD: 485/535, and FLAA: 535/595, respectively.
